# The Vilayam Framework: a conceptual model of bidirectional human development from devolution to flourishing

**DOI:** 10.3389/fpsyg.2026.1916474

**Published:** 2026-09-04

**Authors:** Balram Aatmanayana Rishi

**Affiliations:** Vilayam Institute for Consciousness and Humanitarian Science, Kailash Yaatra Ashram, Demerara, Guyana

**Keywords:** bidirectional development, consciousness, contemplative science, developmental psychology, developmental trajectories, flourishing, human development, hypothesis-generating framework

## Abstract

Contemporary developmental psychology, positive psychology, prevention science, and contemplative research have substantially advanced understanding of human growth, resilience, flourishing, and the factors associated with psychological and behavioural outcomes across the lifespan. However, comparatively less theoretical attention has been devoted to whether the developmental processes that precede observable manifestations constitute a distinct domain of scientific inquiry. This article *introduces the Vilayam Framework, a* conceptual model proposing that human development may be understood as a bidirectional process characterised by movement toward increasing integration or increasing fragmentation while biological life continues. The framework advances three interconnected theoretical propositions. First, it proposes that human development is fundamentally bidirectional rather than exclusively progressive. Second, it introduces *progressive internal unconsciousness while biologically alive* as a proposed developmental construct referring to reductions in conscious participation rather than neurological impairment or psychiatric diagnosis. Third, it proposes the *Journey Before Manifestation* as a developmental domain concerned with investigating developmental trajectories preceding observable psychological, behavioural, relational, educational, social, and physical manifestations. These propositions are integrated within a conceptual architecture describing reciprocal relationships among conscious participation, developmental patterns, narratives, identity, fixation, and manifestation, while emphasising that developmental trajectories remain dynamic, multidetermined, and potentially reversible. The Vilayam Framework is presented as a hypothesis-generating model that complements rather than replaces established developmental theories, diagnostic systems, and evidence-based interventions. Its principal contribution is the proposal that developmental science may benefit from investigating not only observable manifestations and recognised risk factors, but also the developmental trajectories through which manifestations emerge. As a conceptual framework, the model does not claim empirical validation. Instead, it offers theoretically defined propositions intended to stimulate interdisciplinary dialogue, operational development, longitudinal investigation, and empirical evaluation across developmental psychology, prevention science, positive psychology, contemplative science, education, neuroscience, psychiatry, and public health.

## 1 Introduction

### 1.1 Human development beyond a unidirectional perspective

Human development has long been recognized as a dynamic, multidimensional, and lifelong process shaped by the continuous interaction of biological, psychological, developmental, social, cultural, and environmental influences (Baltes et al., 2006; Bronfenbrenner and Morris, 2006; Lerner, 2006; Overton, 2015). Across developmental psychology and the behavioural sciences, extensive theoretical and empirical work has examined how individuals acquire increasingly adaptive capacities for learning, self-regulation, resilience, meaning-making, interpersonal functioning, and psychological wellbeing throughout the lifespan.

Over the past two decades, positive psychology has substantially expanded this understanding by shifting scientific attention beyond psychopathology toward the systematic study of flourishing, character strengths, optimism, resilience, engagement, purpose, and wellbeing (Keyes, 2002; Peterson and Seligman, 2004; Ryff, 1989; Seligman, 2011; Seligman and Csikszentmihalyi, 2000). Similarly, contemplative science has demonstrated that practices cultivating awareness, attention, compassion, ethical engagement, and self-regulation are associated with improvements in psychological functioning and human flourishing (Dahl et al., 2020; Davidson and Dahl, 2018; Lutz et al., 2007; Tang et al., 2015). Together, these fields have contributed significantly to contemporary understanding of how individuals develop toward greater integration, adaptive functioning, and wellbeing.

At the same time, developmental psychology, developmental psychopathology, psychiatry, neuroscience, and public health have advanced scientific understanding of the biological, psychological, and environmental factors associated with psychological disorders, behavioural dysregulation, relational difficulties, and physical illness. These disciplines have generated sophisticated theoretical models explaining developmental risk, vulnerability, resilience, and prevention while also providing increasingly refined diagnostic and intervention frameworks (American Psychiatric Association, 2022; World Health Organization, 2022).

Despite these important advances, one broader conceptual question remains comparatively underexplored.

*Can the developmental processes that precede observable manifestations themselves constitute a distinct domain of scientific inquiry?*

Most existing theoretical and clinical frameworks necessarily concentrate on developmental influences, identifiable risk factors, established patterns of functioning, or observable outcomes. Preventive sciences seek to reduce risk before disorders become established, and developmental theories describe the mechanisms through which individuals grow, adapt, or experience maladaptive outcomes. However, comparatively less attention has been directed toward developing an integrated conceptual framework specifically focused on the developmental progression that may unfold before psychological, behavioural, relational, educational, social, or physical manifestations become sufficiently apparent to attract clinical, educational, or societal attention.

**Figure 1:**
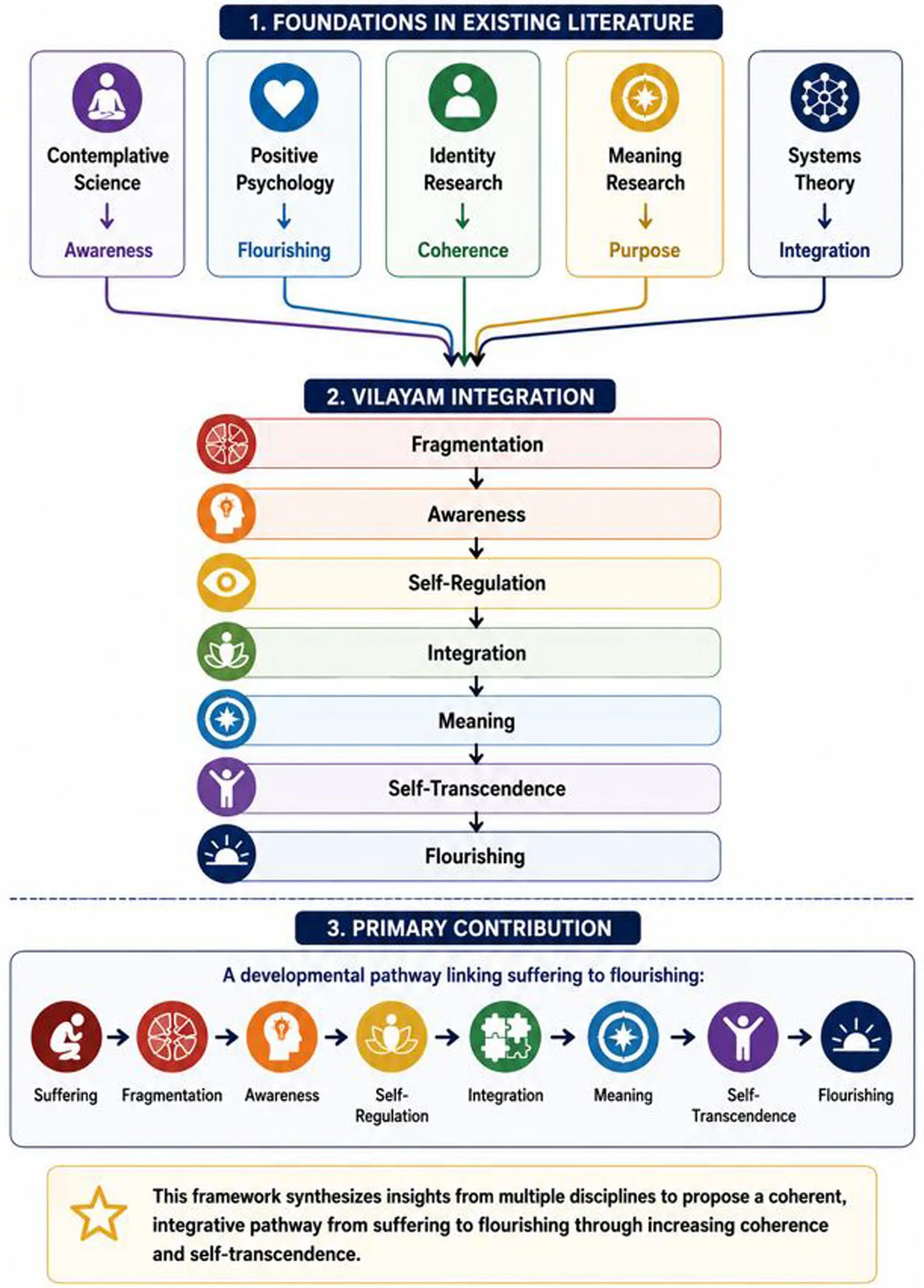
Proposed mechanisms within the Vilayam Framework. Arrows indicate conceptual influence or developmental progression.

**Figure 2:**
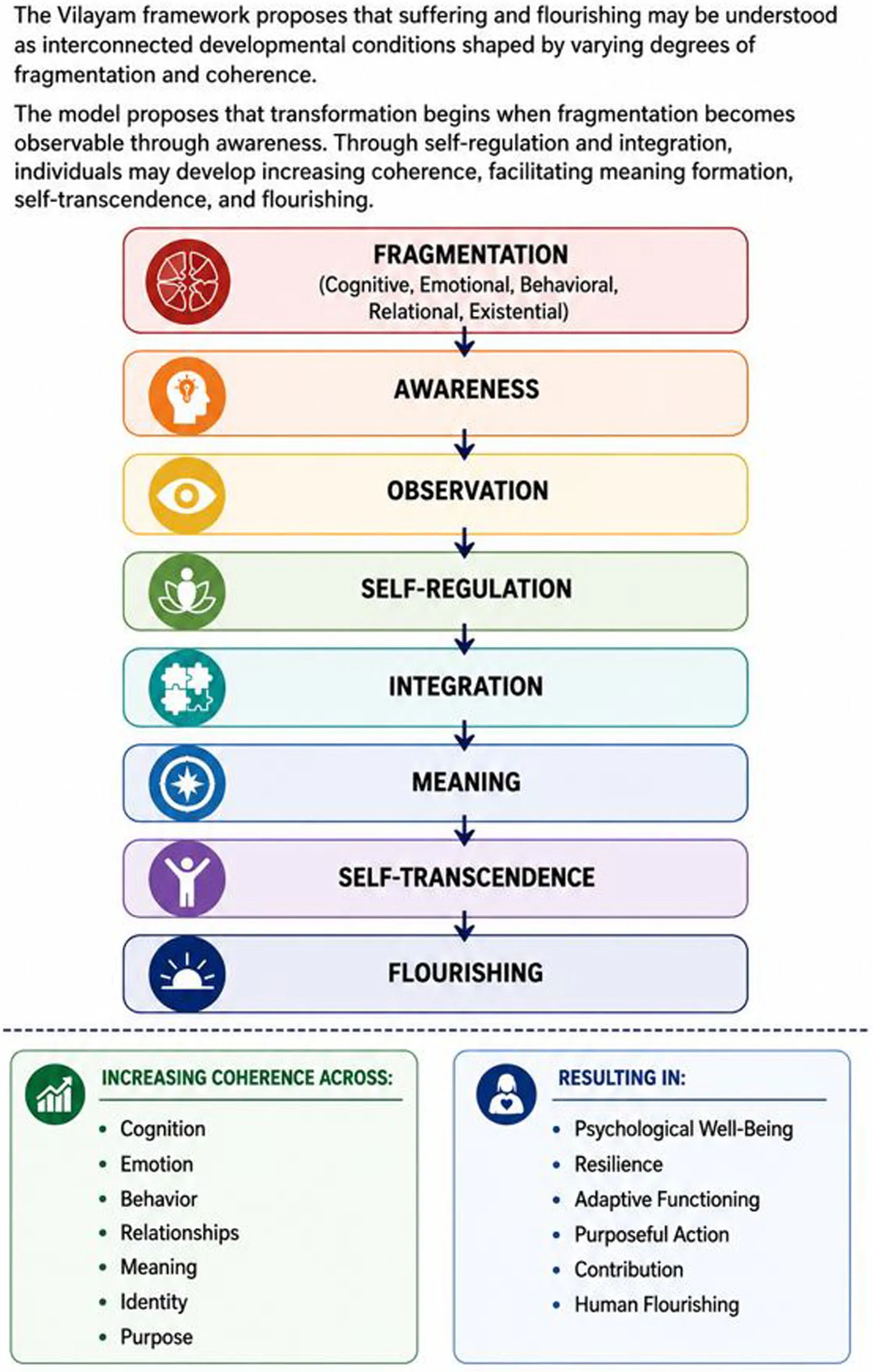
The Vilayam transformation model.

The present manuscript introduces the *Vilayam Framework* as a conceptual contribution intended to address this theoretical space. Rather than replacing established theories within developmental psychology, positive psychology, contemplative science, psychiatry, neuroscience, or public health, the framework proposes an additional developmental perspective through which earlier developmental processes may be conceptualized and investigated (see Figures 1, 2).

The framework advances three interrelated theoretical propositions.

First, it proposes that *human development is fundamentally bidirectional*, encompassing trajectories toward both increasing integration and increasing fragmentation.

Second, it introduces *progressive internal unconsciousness while biologically alive* as a theoretical developmental construct describing gradual reductions in conscious participation that may occur while biological functioning remains intact. This construct is conceptually distinct from neurological disorders of consciousness and is presented as a hypothesis requiring empirical investigation.

Third, it proposes the *Journey Before Manifestation* as a developmental domain for investigating the processes that may precede observable psychological, behavioural, relational, educational, social, or physical manifestations. Within this framework, observable manifestations are understood not necessarily as developmental beginnings, but as potential outcomes of longer developmental trajectories that warrant systematic investigation.

Collectively, these propositions seek to broaden contemporary discussions of human development by extending scientific inquiry beyond observable manifestations toward the developmental processes that may precede them. The framework is presented as a conceptual model designed to generate empirically testable hypotheses, encourage interdisciplinary dialogue, and stimulate future research in developmental psychology, contemplative science, positive psychology, prevention, education, psychiatry, neuroscience, and related disciplines.

The remainder of this paper first presents the framework’s three central theoretical propositions before developing the concepts of bidirectional human development, progressive internal unconsciousness while biologically alive, and the Journey Before Manifestation. It then examines the framework’s integration with contemporary psychological theory, discusses implications for prevention and human flourishing, and concludes by outlining priorities for future empirical investigation.

## 2 Novel theoretical contributions of the Vilayam Framework

The Vilayam Framework is presented as a conceptual contribution to the interdisciplinary study of human development. Rather than proposing an alternative to established theories in developmental psychology, positive psychology, contemplative science, psychiatry, neuroscience, or public health, the framework seeks to expand the scope of developmental inquiry by introducing additional conceptual questions regarding developmental processes that may precede observable manifestations.

Contemporary theories have substantially advanced scientific understanding of human growth, adaptation, resilience, flourishing, developmental risk, and psychopathology (Baltes et al., 2006; Bronfenbrenner and Morris, 2006; Keyes, 2002; Ryan and Deci, 2001; Seligman, 2011). Likewise, preventive science has emphasized the importance of identifying risk and protective factors before disorders become established (Mrazek and Haggerty, 1994). The Vilayam Framework builds upon these contributions while proposing that the developmental progression preceding observable manifestations may itself represent a distinct conceptual focus for scientific investigation.

**Figure 3:**
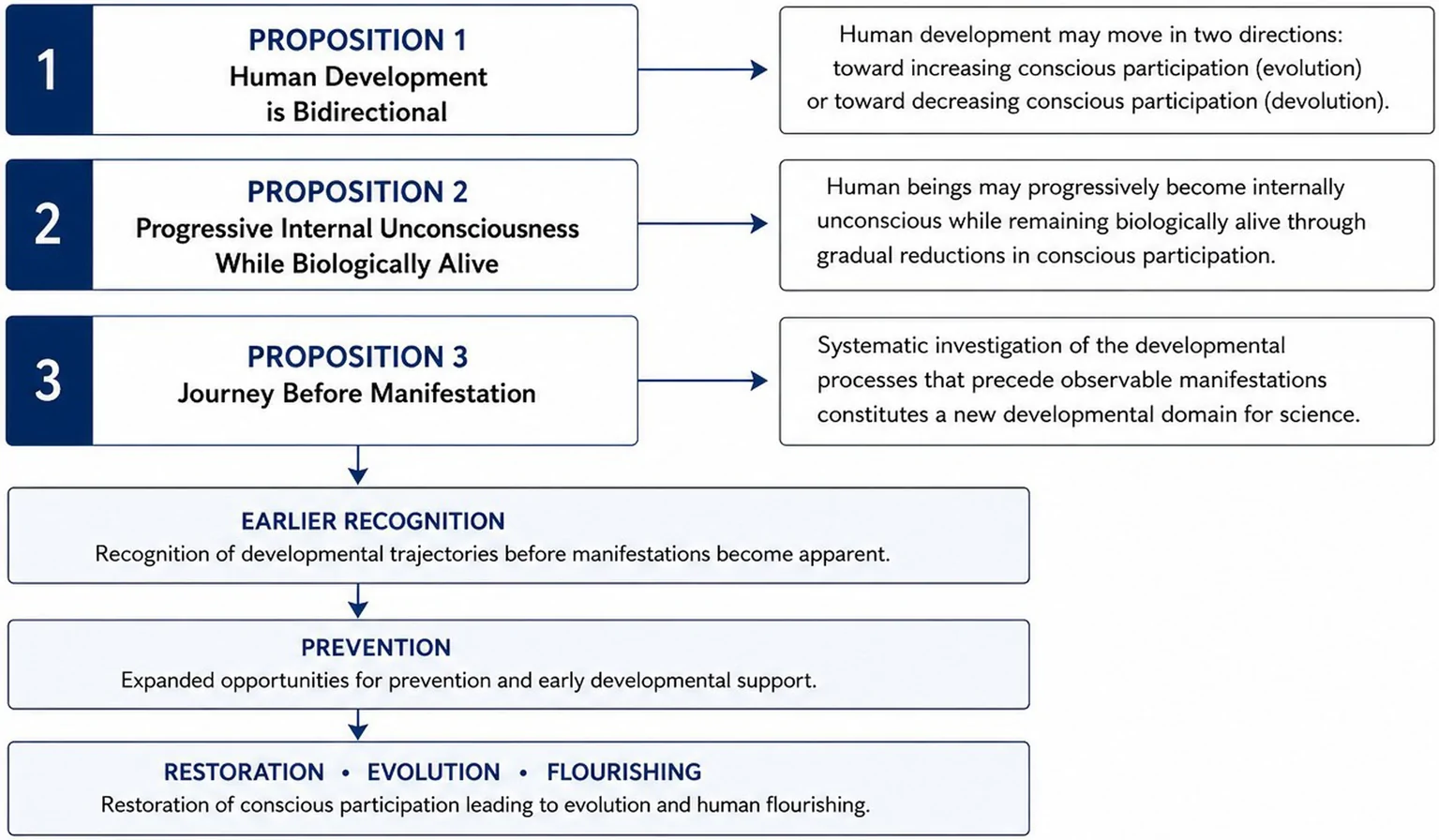
The three theoretical propositions of the Vilayam Framework. The framework advances three interconnected propositions: (1) human development is bidirectional; (2) progressive internal unconsciousness while biologically alive is proposed as a developmental construct; and (3) the journey before manifestation is introduced as a proposed developmental domain for scientific investigation. Together, these propositions provide the conceptual foundation of the framework. The propositions are presented as conceptual contributions intended to inform future empirical research.

**Figure 4:**
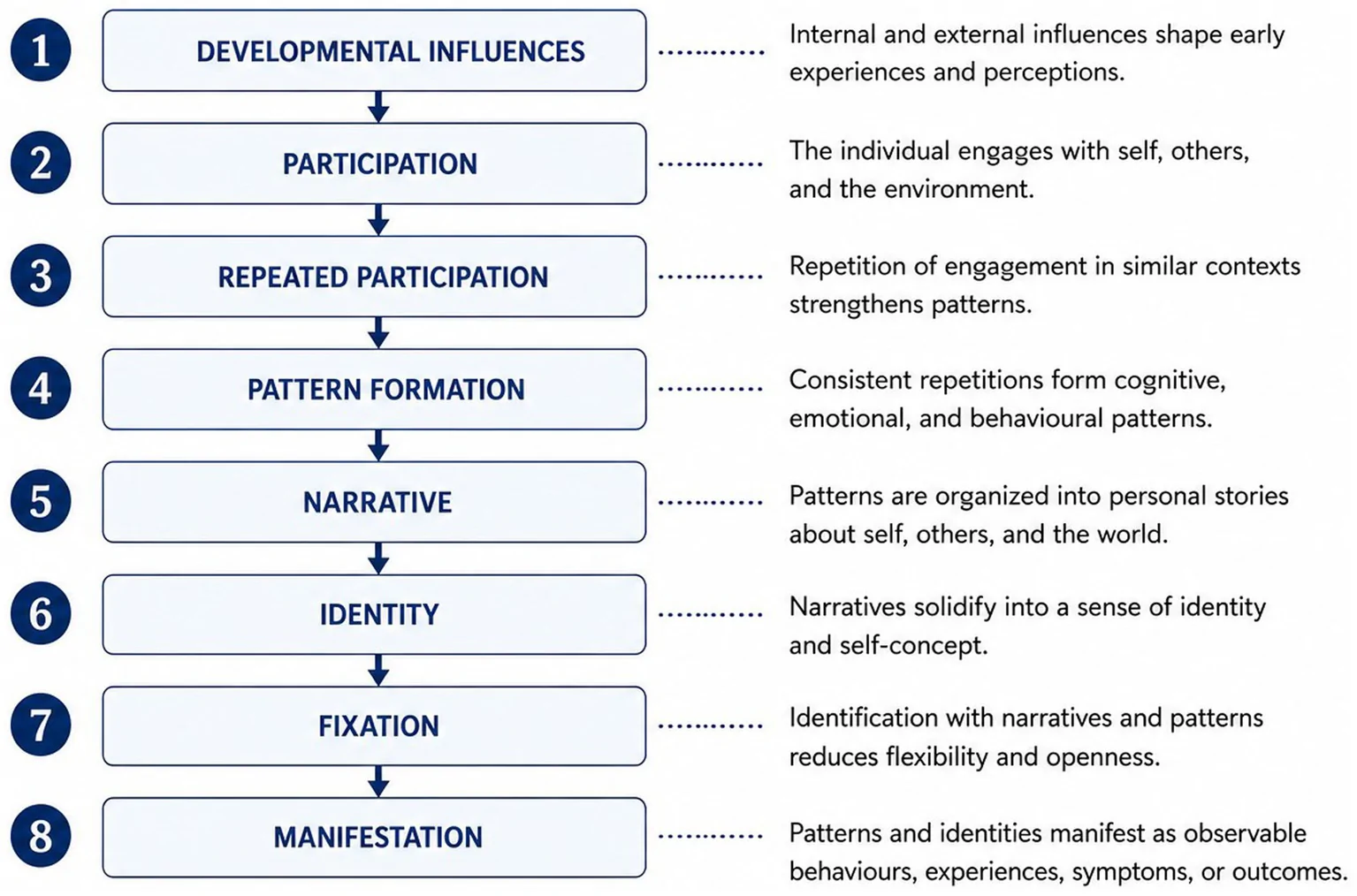
The journey before manifestation. Conceptual illustration of the developmental processes hypothesized to precede observable psychological, behavioural, relational, social, educational, or physical manifestations. The sequence is presented as a theoretical model for future empirical investigation and is not intended to represent a deterministic pathway. Arrows indicate the proposed developmental progression.

**Figure 5:**
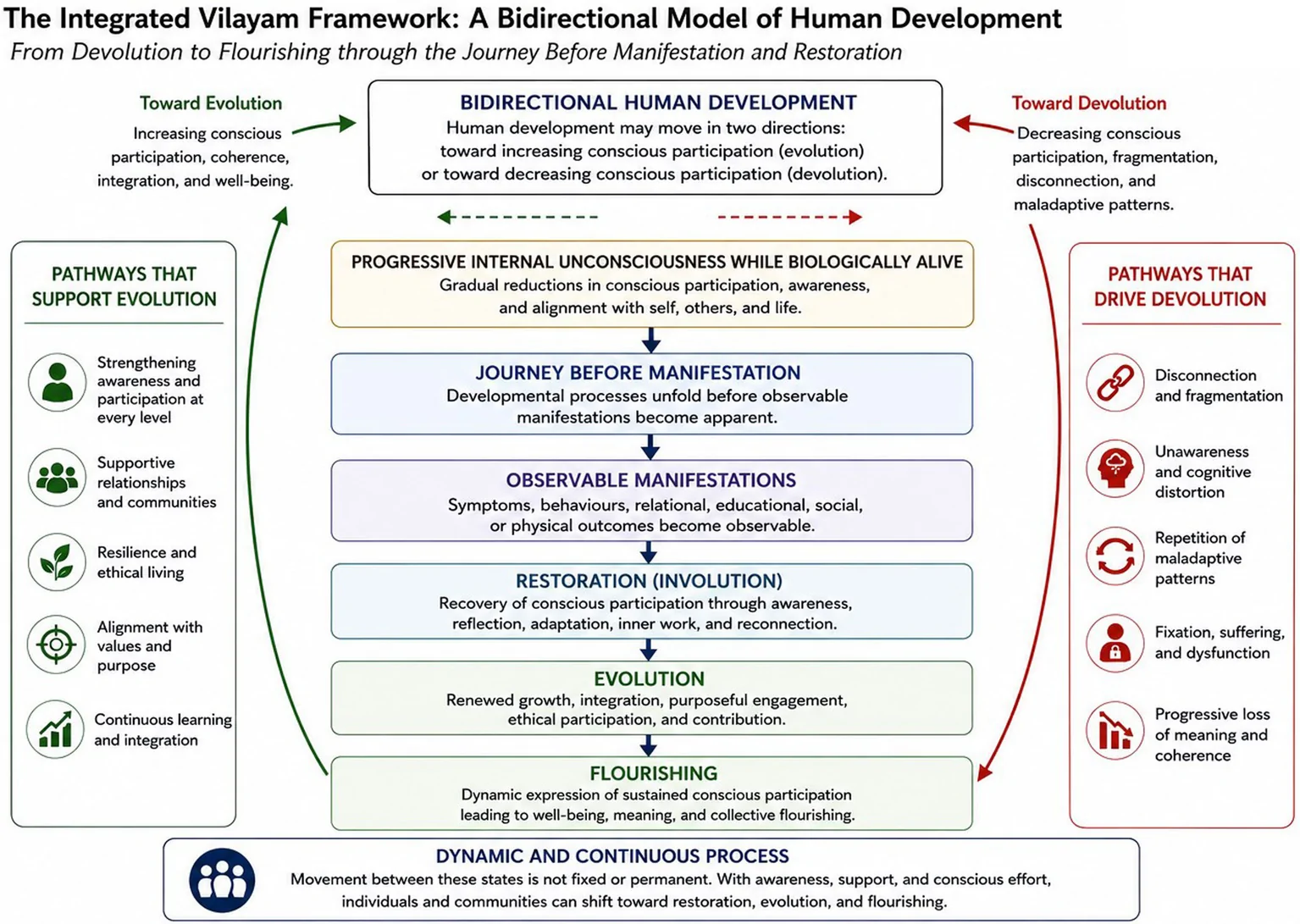
The integrated Vilayam Framework. Conceptual representation of the proposed relationships among bidirectional human development, progressive internal unconsciousness while biologically alive, the Journey Before Manifestation, observable manifestations, restoration (involution), evolution, and flourishing. The model is presented as a conceptual framework designed to generate empirically testable hypotheses.

The framework does not suggest that existing theories are incomplete or inadequate. Rather, it proposes that they may be complemented by a conceptual perspective that explicitly examines developmental movement before many psychological, behavioural, relational, educational, social, or physical outcomes become evident. From this perspective, observable manifestations are understood as potential expressions of longer developmental trajectories rather than necessarily representing the beginning of those trajectories (see Figures 3–5).

Accordingly, the framework advances three interrelated theoretical propositions that together define its conceptual contribution.

### 2.1 Proposition 1: human development is fundamentally bidirectional

The first proposition is that human development is fundamentally bidirectional. Existing developmental theories have extensively examined processes through which individuals acquire increasingly adaptive capacities for learning, self-regulation, resilience, psychological wellbeing, and flourishing (Baltes et al., 2006; Lerner, 2006; Overton, 2015). The Vilayam Framework extends this discussion by proposing that development may also proceed toward increasing fragmentation through progressive reductions in conscious participation while biological life continues.

Importantly, bidirectionality within the present framework does not imply a simple opposition between positive and negative outcomes, nor does it assume that all individuals follow uniform developmental pathways. Rather, it proposes that developmental movement may occur in multiple directions over time, with individuals potentially moving toward increasing integration, increasing fragmentation, or subsequent restoration depending upon complex interactions among biological, psychological, relational, environmental, developmental, cultural, and contextual influences.

### 2.2 Proposition 2: progressive internal unconsciousness while biologically alive

The second proposition introduces progressive internal unconsciousness while biologically alive as a theoretical developmental construct.

Within the Vilayam Framework, this construct refers to gradual reductions in conscious participation while biological functioning remains intact. It is conceptually distinct from neurological disorders of consciousness such as coma, the minimally conscious state, or unresponsive wakefulness syndrome, and it should not be interpreted as a replacement for established neuroscientific or psychological concepts (Gosseries et al., 2014; Laureys et al., 2010).

Instead, the construct is proposed as a developmental hypothesis suggesting that changes in the quality of conscious participation may unfold progressively across time before many observable manifestations become apparent. As presented in this manuscript, the construct remains theoretical and requires operational definition, empirical investigation, and validation through future interdisciplinary research.

### 2.3 Proposition 3: the journey before manifestation

Building upon the previous propositions, the framework introduces the Journey Before Manifestation as a proposed developmental domain.

Whereas much existing research necessarily focuses upon identifiable symptoms, behaviours, disorders, or established outcomes, the Journey Before Manifestation directs attention toward developmental processes that may occur before such manifestations become clinically, educationally, behaviourally, or socially observable.

The Journey Before Manifestation is not proposed as a diagnostic framework, nor does it seek to replace existing approaches to prevention, assessment, or intervention. Rather, it provides a conceptual domain through which developmental movement may be examined longitudinally, generating hypotheses concerning earlier developmental change and encouraging interdisciplinary investigation across psychology, education, neuroscience, public health, contemplative science, and related disciplines.

### 2.4 Conceptual integration of the three propositions

Although each proposition can be considered independently, their principal contribution lies in their integration.

The proposition of bidirectional human development establishes the broader developmental architecture. The construct of progressive internal unconsciousness while biologically alive provides a theoretical account of one possible developmental trajectory within that architecture. The Journey Before Manifestation identifies the proposed domain through which these developmental trajectories may be investigated before observable manifestations become established.

Taken together, these propositions define the Vilayam Framework as a conceptual model that expands the temporal scope of developmental inquiry. Rather than asking only why observable manifestations occur, the framework additionally asks whether the developmental movement preceding those manifestations can itself be systematically described, operationalized, measured, and investigated through interdisciplinary research.

Accordingly, the present paper should be understood as introducing a research programme rather than presenting established empirical conclusions. The purpose of the framework is to generate scientifically testable hypotheses capable of supporting future theoretical refinement, empirical evaluation, and interdisciplinary dialogue.

## 3 Human development as a bidirectional process

### 3.1 A theoretical proposition

Contemporary theories of human development have substantially advanced scientific understanding of the processes through which individuals grow, adapt, regulate behaviour, develop resilience, cultivate meaning, and achieve psychological wellbeing across the lifespan (Baltes et al., 2006; Bronfenbrenner and Morris, 2006; Lerner, 2006; Overton, 2015). Positive psychology and contemplative science have further demonstrated that capacities such as awareness, psychological flexibility, compassion, self-regulation, ethical engagement, and reflective functioning contribute to adaptive development and human flourishing (Davidson and Dahl, 2018; Ryan and Deci, 2001; Seligman, 2011; Tang et al., 2015).

These perspectives have collectively strengthened scientific understanding of developmental processes that support increasingly adaptive human functioning. However, comparatively less theoretical attention has been devoted to whether development itself may be conceptualized as movement in more than one direction across the lifespan.

The Vilayam Framework proposes that human development is fundamentally bidirectional.

Within this framework, bidirectionality refers to the possibility that developmental movement may occur toward increasing integration or toward increasing fragmentation through changes in conscious participation while biological life continues. The framework therefore conceptualizes development not as a single trajectory of progressive growth, but as a dynamic developmental system in which multiple trajectories remain possible throughout life.

Importantly, this proposition should not be interpreted as suggesting that development proceeds along two fixed or opposing pathways. Human development remains multidetermined and continuously influenced by complex interactions among biological, psychological, developmental, relational, environmental, cultural, educational, and socioeconomic factors (Baltes et al., 2006; Bronfenbrenner and Morris, 2006; Overton, 2015). Individuals may move toward greater integration, experience periods of fragmentation, recover through restoration, or demonstrate different developmental patterns across multiple domains simultaneously.

Accordingly, bidirectionality is understood as a property of developmental movement rather than as a classification of persons.

Within the Vilayam Framework, increasing integration is associated with progressively greater conscious participation, adaptive self-regulation, reflective awareness, meaningful relationships, ethical engagement, and coherent participation in life. Conversely, increasing fragmentation refers to the progressive reduction of these integrative capacities through diminished conscious participation while biological functioning continues.

The framework does not propose that fragmentation represents pathology, moral failure, or an inevitable precursor to disorder. Nor does it suggest that every manifestation of psychological distress, behavioural dysregulation, or physical illness follows a single developmental pathway. Rather, fragmentation is proposed as one possible developmental trajectory whose characteristics, mechanisms, and outcomes require empirical investigation.

From this perspective, observable manifestations are understood as potential outcomes of longer developmental processes rather than necessarily representing the beginning of those processes. Consequently, developmental inquiry may be expanded by asking not only *what manifestation is present*, but also *what developmental trajectory preceded its emergence*.

This proposition has important implications for developmental science.

If developmental trajectories can move toward increasing or decreasing conscious participation, then the direction of developmental movement itself becomes an important object of scientific investigation. Research may therefore examine whether developmental change can be recognized before manifestations become clinically, behaviourally, educationally, socially, or physically apparent, thereby extending opportunities for earlier recognition, prevention, restoration, and the promotion of human flourishing.

The present manuscript does not claim that bidirectional human development has been empirically established. Rather, it introduces the proposition as a conceptual hypothesis intended to stimulate interdisciplinary research. Longitudinal investigation, psychometric development, comparative theoretical analysis, and prospective empirical studies will be necessary to determine whether the proposed trajectories can be operationalized, measured, differentiated from existing constructs, and evaluated scientifically.

Accordingly, bidirectional human development should be understood as the conceptual foundation upon which the remaining elements of the Vilayam Framework are constructed. The propositions of progressive internal unconsciousness while biologically alive and the Journey Before Manifestation derive directly from this broader developmental architecture and are presented as complementary theoretical constructs requiring future empirical investigation.

## 4 The journey before manifestation: a proposed developmental domain

Contemporary psychology, psychiatry, neuroscience, education, public health, and related disciplines have made substantial advances in understanding, classifying, and treating observable manifestations of human functioning. Psychological symptoms, behavioural dysregulation, addictive behaviours, relational difficulties, educational challenges, and physical illness have been investigated through increasingly sophisticated theoretical, diagnostic, and intervention frameworks (American Psychiatric Association, 2022; Mrazek and Haggerty, 1994; World Health Organization, 2022). These contributions remain fundamental to contemporary science and clinical practice.

The Vilayam Framework does not seek to replace these established approaches.

Instead, it proposes an additional developmental question:

*Can the developmental processes that precede observable manifestations themselves constitute a distinct domain of scientific inquiry?*

This question forms the conceptual basis of what the framework terms the *Journey Before Manifestation*.

Within the Vilayam Framework, the Journey Before Manifestation is proposed as a developmental domain concerned with investigating the progression of developmental processes that may occur before observable psychological, behavioural, relational, educational, social, or physical manifestations become evident. The emphasis is not on replacing existing explanations of manifestation, but on extending scientific inquiry further upstream in the developmental process.

This proposal differs from asking simply whether risk factors exist. Risk factors have long been recognised within developmental psychology, psychiatry, and public health. Rather, the Journey Before Manifestation proposes that the progression linking developmental influences to later manifestations may itself be conceptualised, described, and investigated as a coherent developmental domain.

Within this perspective, manifestations are understood not as isolated events but as possible outcomes of longer developmental trajectories. Consequently, scientific attention expands beyond questions such as:

- What diagnosis is present?
- What symptoms are observable?
- What behaviour requires intervention?

to include an additional developmental question:

*What developmental journey preceded the manifestation?*

This shift represents an expansion of developmental inquiry rather than a redefinition of diagnosis. Diagnostic systems remain essential for identifying and managing established conditions, while the Journey Before Manifestation seeks to investigate developmental processes that may occur before such conditions become clinically, behaviourally, educationally, or socially recognisable.

Importantly, the framework does not propose that all manifestations arise through identical developmental pathways, nor does it imply that every manifestation is preceded by a single universal process. Human development remains influenced by complex interactions among biological, psychological, developmental, relational, environmental, cultural, educational, and socioeconomic factors. Accordingly, the Journey Before Manifestation is proposed as a conceptual domain capable of accommodating multiple developmental trajectories rather than prescribing one deterministic sequence.

This perspective has important implications for prevention.

Traditional prevention often focuses on reducing identifiable risks before disorders become established. The Journey Before Manifestation complements these approaches by proposing that earlier developmental movement itself may represent an additional opportunity for investigation and intervention. If developmental trajectories can be recognised before observable manifestations emerge, preventive strategies may be informed not only by recognised risk factors but also by patterns of developmental change occurring across time.

The Journey Before Manifestation also has implications for interdisciplinary research. Because developmental movement involves interacting biological, psychological, relational, environmental, educational, contemplative, and social influences, its investigation requires collaboration across multiple disciplines rather than reliance upon any single explanatory model. Developmental psychology, neuroscience, psychiatry, education, sociology, public health, and contemplative science each contribute complementary perspectives capable of informing the study of developmental progression before manifestation.

As presented in this manuscript, the Journey Before Manifestation is not an empirical finding but a conceptual proposal. Future research will be required to determine whether developmental trajectories preceding observable manifestations can be operationalised, measured, distinguished from existing constructs, and evaluated through longitudinal and interdisciplinary investigation.

Accordingly, the Journey Before Manifestation should be understood as the principal developmental domain proposed by the Vilayam Framework. Its purpose is to generate new scientific questions regarding the developmental progression preceding manifestation while complementing existing approaches to prevention, diagnosis, intervention, restoration, and the promotion of human flourishing.

## 5 Progressive internal unconsciousness while biologically alive: the central theoretical proposition

The central theoretical proposition of the Vilayam Framework is that biological life and conscious participation should not necessarily be regarded as equivalent constructs. While human beings may remain biologically alive and capable of carrying out many ordinary activities, the quality of their conscious participation in their own developmental processes may vary across time. The framework proposes that this variation represents a meaningful dimension of human development deserving systematic scientific investigation.

Within contemporary neuroscience, disorders of consciousness refer to clinically identifiable conditions involving impaired wakefulness or awareness, including coma, the minimally conscious state, and unresponsive wakefulness syndrome (Gosseries et al., 2014; Laureys et al., 2010). These conditions are characterised by measurable neurological dysfunction and remain the subject of extensive clinical research.

The Vilayam Framework does not address these neurological conditions.

Instead, it introduces *progressive internal unconsciousness while biologically alive* as a proposed developmental construct.

Within the present framework, progressive internal unconsciousness refers to the gradual reduction in *conscious participation* while biological functioning remains intact. It does not imply loss of wakefulness, neurological impairment, diminished intelligence, or the psychoanalytic unconscious. Nor should it be interpreted as a psychiatric diagnosis or a clinical disorder.

Rather, it is proposed as a developmental hypothesis suggesting that individuals may progressively lose awareness of how they participate in the ongoing processes through which patterns of thought, behaviour, relationships, identity, and lived experience are continually formed and reinforced.

The emphasis of the construct is therefore not on whether a person is conscious in the neurological sense, but on the quality of participation in the developmental processes that shape everyday functioning.

Within the Vilayam Framework, conscious participation refers to the capacity to engage intentionally, reflectively, adaptively, and responsibly with one’s thoughts, emotions, behaviours, relationships, values, decisions, and broader life context. Progressive internal unconsciousness proposes that this capacity may gradually diminish across time while biological functioning continues uninterrupted.

This proposition differs conceptually from several established constructs. It is distinct from neurological disorders of consciousness, which concern impaired wakefulness or awareness resulting from identifiable neurological injury. It is also distinct from the psychoanalytic unconscious, which describes mental processes operating outside conscious awareness. Likewise, it is not synonymous with psychological inflexibility, emotional dysregulation, maladaptive coping, or deficits in self-regulation, although future empirical research may investigate areas of conceptual overlap and divergence.

Accordingly, progressive internal unconsciousness should be understood as a proposed organising construct rather than a replacement for existing psychological theories. Its purpose is to provide a conceptual framework through which developmental reductions in conscious participation may be investigated alongside established explanations of human functioning.

The introduction of this construct has an important theoretical implication.

If conscious participation can progressively diminish while biological functioning remains intact, then many observable manifestations may represent relatively late expressions of longer developmental trajectories rather than their point of origin. From this perspective, the developmental progression preceding manifestation becomes an important object of scientific inquiry, providing the conceptual basis for the Journey Before Manifestation introduced in the previous section.

The present manuscript does not claim that progressive internal unconsciousness has been empirically demonstrated. Rather, it proposes the construct as a theoretical hypothesis requiring careful operational definition and systematic investigation. Future research will be needed to determine whether it can be distinguished from related psychological constructs, measured reliably, examined longitudinally, and evaluated across diverse populations and developmental contexts.

If supported through empirical investigation, the construct may contribute to a broader understanding of how developmental change unfolds before many observable manifestations become clinically, behaviourally, educationally, or socially apparent. If not supported, the framework should be revised accordingly. In this respect, progressive internal unconsciousness is presented not as a conclusion but as a scientifically testable proposition intended to stimulate interdisciplinary dialogue and future research.

## 6 Proposed developmental mechanisms of bidirectional human development

Having proposed that human development is fundamentally bidirectional and that progressive internal unconsciousness while biologically alive represents one possible developmental trajectory, the Vilayam Framework further proposes that developmental movement occurs through interacting developmental processes rather than isolated events. The framework does not propose a single causal pathway. Instead, it presents a conceptual architecture in which biological, psychological, developmental, relational, environmental, educational, cultural, and contemplative influences continuously interact throughout the lifespan, shaping the direction and quality of human development (Baltes et al., 2006; Bronfenbrenner and Morris, 2006; Overton, 2015).

Within this conceptual architecture, the central organising process is *conscious participation*.

Participation refers to the ongoing manner in which individuals engage with their thoughts, emotions, behaviours, relationships, values, environments, and lived experiences. It is not viewed as a discrete behaviour or isolated mental state, but as a continuous developmental process through which individuals actively shape, and are simultaneously shaped by, their interactions with themselves and the world around them (Bandura, 1986; Carver and Scheier, 1998; Ryan and Deci, 2001).

The framework proposes that repeated forms of participation gradually give rise to *developmental patterns*.

Patterns emerge through the repeated organisation of thoughts, emotions, behaviours, and relational responses across time. As these patterns become increasingly stable, they provide a degree of predictability and continuity in how individuals respond to similar situations. At this stage, development remains flexible, and patterns may strengthen, weaken, or change in response to new experiences and environments.

Repeated patterns gradually contribute to the formation of *developmental narratives*.

Narratives represent the interpretive frameworks through which individuals make sense of themselves, other people, and their lived experiences. These narratives organise experience into coherent explanations that influence expectations, beliefs, motivations, and future participation. Importantly, narratives are not passive reflections of experience; they also shape subsequent participation by influencing how future situations are interpreted.

Over time, repeatedly reinforced narratives contribute to the development of *identity*.

Identity refers to the relatively stable organisation of self-understanding that emerges through continuous interaction between participation, experience, and interpretation. Rather than existing independently, identity is continuously influenced by ongoing participation while simultaneously shaping future participation through reciprocal feedback. Consequently, identity within the Vilayam Framework is understood as a dynamic developmental process rather than a fixed personal characteristic.

As patterns and identities become increasingly reinforced, developmental flexibility may progressively diminish.

The framework refers to this proposed process as *fixation*.

Fixation does not imply permanence or pathology. Rather, it describes the progressive stabilisation of developmental patterns that become increasingly resistant to adaptive modification. The greater the degree of fixation, the more developmental movement may become self-reinforcing through reciprocal interactions among participation, patterns, narratives, and identity.

Observable manifestations are proposed to emerge within this broader developmental context.

Manifestations—including psychological symptoms, behavioural dysregulation, relational difficulties, educational challenges, or physical illness—are therefore conceptualised as possible expressions of extended developmental trajectories rather than isolated developmental beginnings. Importantly, manifestations are not viewed as inevitable consequences of fixation, nor does the framework propose that every manifestation follows this sequence. Instead, the proposed architecture provides one conceptual model through which developmental progression may be organised and investigated.

Accordingly, the Vilayam Framework proposes the following conceptual sequence:

*Participation ↔ Pattern Formation ↔ Narrative ↔ Identity ↔ Fixation → Manifestation.*

The use of reciprocal relationships reflects the framework’s position that developmental processes continuously influence one another. Participation shapes narratives, while narratives simultaneously influence future participation. Identity both emerges from and contributes to participation. Likewise, fixation is proposed to strengthen existing developmental organisation while remaining theoretically reversible through subsequent developmental change.

Within this architecture, movement toward increasing integration is proposed to occur when participation progressively strengthens reflective awareness, adaptive self-regulation, ethical engagement, psychological flexibility, meaningful relationships, and coherent participation in life. Conversely, movement toward increasing fragmentation is proposed to occur when participation progressively weakens these integrative capacities through reductions in conscious participation while biological functioning remains intact.

Importantly, this conceptual architecture should not be interpreted as deterministic or universally applicable. Individual developmental trajectories remain influenced by numerous interacting biological, psychological, relational, environmental, educational, cultural, and contextual factors. The proposed mechanisms therefore represent a heuristic model intended to organise developmental inquiry rather than a definitive account of all human development.

Future empirical research will be required to determine whether these proposed developmental mechanisms can be operationalised, observed longitudinally, differentiated from related constructs, and evaluated across diverse developmental contexts. Such investigations will determine whether the conceptual architecture proposed by the Vilayam Framework contributes meaningfully to contemporary developmental science.

## 7 Developmental trajectories: devolution, restoration, and evolution

The Vilayam Framework proposes that bidirectional human development may be expressed through three broad developmental trajectories: *devolution*, *restoration*, and *evolution*. These trajectories are not presented as fixed stages through which every individual must progress, nor should they be interpreted as diagnostic categories. Rather, they represent conceptual descriptions of possible directions of developmental movement that may occur across the lifespan.

Human development is understood as dynamic, multidirectional, and continuously responsive to biological, psychological, relational, environmental, cultural, educational, and contextual influences (Baltes et al., 2006; Bronfenbrenner and Morris, 2006; Overton, 2015). Consequently, individuals may move toward greater integration, experience periods of fragmentation, recover through restoration, or demonstrate different developmental trajectories simultaneously across different domains of life.

Within this framework, *devolution* refers to a proposed developmental trajectory characterised by the progressive reduction of conscious participation while biological life continues. It is conceptualised as movement toward increasing fragmentation in the ways individuals engage with themselves, their relationships, their environments, and their lived experiences.

Importantly, devolution is *not* presented as moral decline, personal failure, spiritual deficiency, or psychiatric diagnosis. Rather, it is proposed as one possible developmental direction through which adaptive participation may gradually diminish across time. The concept therefore serves as a developmental descriptor rather than a judgment about individuals.

The framework further proposes that developmental movement is not necessarily irreversible.

The second trajectory, *restoration*, describes the gradual recovery of conscious participation through increasing reflective awareness, adaptive engagement, self-regulation, meaningful relationships, and coherent participation in life. Restoration corresponds to what the Vilayam Framework traditionally refers to as *involution*, but the present manuscript adopts the term *restoration* because it communicates the developmental process more clearly within contemporary psychological literature.

Restoration is not viewed as a single event or isolated intervention. Rather, it is conceptualised as an ongoing developmental process through which previously fragmented patterns may become progressively reorganised. This process may involve strengthening reflective capacity, increasing psychological flexibility, improving adaptive participation, and enhancing meaningful engagement with one’s social and environmental contexts.

The framework further proposes that sustained restoration may contribute to *evolution*.

Within the Vilayam Framework, evolution refers to continued developmental movement toward increasing integration, coherent participation, psychological flexibility, adaptive functioning, meaningful relationships, ethical responsibility, and human flourishing. Evolution is therefore conceptualised not simply as the absence of distress but as the progressive development of capacities that support sustained wellbeing and constructive participation in families, communities, and society (Keyes, 2002; Peterson and Seligman, 2004; Ryan and Deci, 2001; Seligman, 2011; VanderWeele, 2017).

Importantly, evolution is *not* presented as a permanent developmental endpoint.

The framework proposes that human development remains dynamic throughout life. Individuals who have developed adaptive capacities may subsequently encounter circumstances that alter their developmental trajectories, while individuals experiencing fragmentation may move toward increasing integration through restoration. Consequently, developmental movement remains open rather than fixed.

This dynamic perspective distinguishes the proposed trajectories from rigid stage models.

Rather than progressing through predetermined levels, individuals are understood to move continuously in response to changing biological, psychological, relational, environmental, educational, and cultural conditions. Development therefore involves ongoing adaptation rather than irreversible progression.

Within this conceptualisation, devolution, restoration, and evolution should be understood as interconnected developmental trajectories rather than discrete categories. Their significance lies in describing the direction of developmental movement rather than assigning individuals to permanent developmental states.

This perspective has important implications for prevention and human flourishing.

If developmental movement remains reversible, then opportunities for prevention and restoration may exist throughout the lifespan rather than only after observable manifestations have become established. Likewise, flourishing may be understood not as a fixed achievement but as the continuing expression of sustained conscious participation requiring ongoing adaptation, reflection, and engagement.

As with the other constructs introduced in this paper, these developmental trajectories are presented as conceptual propositions rather than empirically established conclusions. Future longitudinal and interdisciplinary research will be required to examine whether these trajectories can be operationalised, differentiated, measured, and evaluated across diverse populations and developmental contexts.

Accordingly, the concepts of devolution, restoration, and evolution provide the dynamic developmental context within which the remaining elements of the Vilayam Framework may be understood. Together they reinforce the framework’s central proposition that human development is an ongoing process characterised by multiple potential directions rather than a single predetermined pathway.

## 8 Relationship to existing developmental and psychological theories

The Vilayam Framework is presented as a complementary conceptual model situated within the broader landscape of developmental science, psychology, psychiatry, neuroscience, public health, and contemplative research. It does not seek to replace established theories of human development, existing diagnostic systems, or evidence-based interventions. Rather, it proposes an additional conceptual perspective through which developmental processes preceding observable manifestations may be investigated.

Contemporary developmental theories have substantially advanced understanding of human growth through ecological, lifespan, systems, cognitive, social, and developmental perspectives (Baltes et al., 2006; Bronfenbrenner and Morris, 2006; Lerner, 2006; Overton, 2015). Likewise, positive psychology has expanded scientific inquiry beyond psychopathology by investigating resilience, strengths, meaning, flourishing, psychological flexibility, and wellbeing (Keyes, 2002; Peterson and Seligman, 2004; Ryan and Deci, 2001; Seligman, 2011; VanderWeele, 2017). Contemplative science has further demonstrated that attention, awareness, compassion, emotional regulation, and contemplative practices may influence adaptive psychological functioning and neuroplasticity (Davidson and Dahl, 2018; Tang et al., 2015).

The Vilayam Framework builds upon these developments while introducing three related conceptual propositions.

First, it proposes that human development may be understood as fundamentally bidirectional, permitting movement toward increasing integration or increasing fragmentation throughout the lifespan.

Second, it introduces progressive internal unconsciousness while biologically alive as a proposed developmental construct concerned with variations in conscious participation rather than neurological consciousness or psychiatric diagnosis.

Third, it proposes the Journey Before Manifestation as a conceptual developmental domain for investigating developmental trajectories preceding observable psychological, behavioural, educational, relational, social, and physical manifestations.

These propositions are intended to extend rather than contradict existing theoretical perspectives.

For example, ecological systems theory emphasises the reciprocal influence of individuals and their environments across multiple developmental contexts (Bronfenbrenner and Morris, 2006). The Vilayam Framework similarly recognises that developmental movement is continuously influenced by interacting biological, psychological, relational, environmental, educational, cultural, and socioeconomic factors. However, it adds the question of whether changes in conscious participation may themselves represent an additional dimension of developmental inquiry.

Similarly, positive psychology has demonstrated that flourishing involves more than the absence of illness by examining positive functioning, meaning, engagement, strengths, and wellbeing (Seligman, 2011; VanderWeele, 2017). The Vilayam Framework is consistent with this perspective while proposing that flourishing may also be understood in relation to the developmental trajectory through which increasing conscious participation is cultivated and maintained.

The framework also aligns with contemporary prevention science, which seeks to identify opportunities for intervention before disorders become established (Mrazek and Haggerty, 1994; World Health Organization, 2022). However, rather than focusing exclusively on identifiable risk factors, the Vilayam Framework proposes that the developmental progression itself may represent an additional object of scientific investigation.

Importantly, the framework does not claim conceptual exclusivity.

Many of its constituent ideas—including self-regulation, reflective awareness, adaptive participation, developmental plasticity, resilience, environmental influence, and human flourishing—have been investigated extensively across multiple disciplines. The proposed contribution lies not in introducing entirely new psychological processes but in organising these processes within an integrated conceptual architecture centred on developmental direction, conscious participation, and the Journey Before Manifestation.

Accordingly, the Vilayam Framework should be understood as a hypothesis-generating model. Its value will ultimately depend not upon conceptual novelty alone but upon whether its propositions can be operationalised, empirically investigated, distinguished from existing constructs, and shown to contribute meaningfully to scientific understanding.

Future comparative research may therefore examine areas of convergence and divergence between the Vilayam Framework and established developmental, psychological, neuroscientific, psychiatric, educational, and contemplative theories. Such investigations will determine whether the framework provides explanatory value beyond existing conceptual models or whether its constructs are more appropriately understood as alternative descriptions of already recognised developmental processes.

In this respect, the Vilayam Framework is offered not as a replacement paradigm but as an invitation to interdisciplinary dialogue. Its principal contribution is the proposal that developmental science may benefit from investigating not only observable manifestations and their associated risk factors but also the developmental trajectories through which such manifestations may emerge.

## 9 Discussion

The Vilayam Framework has been presented as a conceptual model proposing that human development may be understood as a bidirectional process involving movement toward increasing integration or increasing fragmentation throughout the lifespan. Rather than challenging established developmental theories, diagnostic systems, or evidence-based interventions, the framework seeks to expand the scope of developmental inquiry by introducing three interconnected propositions: (1) human development is fundamentally bidirectional; (2) progressive internal unconsciousness while biologically alive represents a proposed developmental construct centred on conscious participation; and (3) the Journey Before Manifestation constitutes a proposed developmental domain concerned with processes preceding observable manifestations.

The principal contribution of the framework is therefore conceptual rather than empirical.

Its purpose is not to demonstrate that these propositions have already been established but to formulate them with sufficient clarity that they may be examined through interdisciplinary scientific investigation. In this respect, the manuscript aligns with the objectives of a Hypothesis & Theory article by generating testable questions capable of informing future research.

One implication of the framework is a shift in the primary focus of developmental investigation.

Much contemporary research understandably concentrates on observable manifestations, including symptoms, disorders, behavioural dysregulation, educational difficulties, relational challenges, and physical illness. The Vilayam Framework proposes that scientific inquiry may also benefit from investigating developmental movement occurring before such manifestations become clinically or socially apparent. If developmental trajectories preceding manifestation can be identified, operationalised, and measured, they may provide additional opportunities for prevention, restoration, and the promotion of human flourishing.

The framework also contributes to ongoing discussions regarding prevention science.

Existing preventive approaches have demonstrated the importance of identifying risk and protective factors throughout development (Mrazek and Haggerty, 1994; World Health Organization, 2022). The Vilayam Framework complements these approaches by proposing that developmental progression itself may represent an additional object of investigation. Rather than asking only which factors increase or decrease risk, researchers may also investigate how developmental movement unfolds across time before manifestations emerge.

Similarly, the framework contributes to contemporary discussions of flourishing.

Positive psychology has established that flourishing encompasses strengths, meaning, resilience, purpose, engagement, and psychological wellbeing (Keyes, 2002; Peterson and Seligman, 2004; Seligman, 2011; VanderWeele, 2017). The Vilayam Framework extends this perspective by proposing that flourishing may also be understood as the continuing expression of adaptive conscious participation within an ongoing developmental trajectory. From this perspective, flourishing is not regarded as a fixed endpoint but as a dynamic developmental process requiring continuous adaptation and engagement.

Importantly, the framework should be interpreted with appropriate caution.

The constructs of progressive internal unconsciousness while biologically alive, conscious participation, the Journey Before Manifestation, and the proposed developmental trajectories have not yet been operationalised or empirically validated. Their conceptual boundaries require further clarification, and future research will be necessary to distinguish them from related constructs within developmental psychology, neuroscience, psychiatry, prevention science, contemplative research, and positive psychology.

The framework therefore has several limitations.

First, it is conceptual rather than empirical and does not present direct experimental evidence supporting its propositions.

Second, several constructs introduced in the framework require operational definitions capable of supporting reliable measurement and longitudinal investigation.

Third, the relationships among the proposed constructs remain theoretical and require empirical examination using diverse methodologies and populations.

Fourth, additional comparative work will be required to determine whether the framework contributes explanatory value beyond existing developmental theories or whether aspects of the model overlap substantially with already established constructs.

These limitations should not be regarded as weaknesses unique to the Vilayam Framework but as characteristic of the early development of conceptual models within scientific inquiry. The advancement of any theoretical framework ultimately depends upon systematic empirical evaluation rather than conceptual argument alone.

Accordingly, several research priorities emerge from the present work.

Future studies may focus on:

- developing operational definitions for conscious participation and progressive internal unconsciousness;
- constructing psychometrically sound measurement instruments;
- conducting longitudinal studies examining developmental trajectories across time;
- comparing the framework with existing developmental, psychological, and neuroscientific models;
- investigating interdisciplinary applications within education, mental health, public health, contemplative science, and community-based prevention.

Ultimately, the scientific value of the Vilayam Framework will depend upon whether its propositions can be operationalised, tested, replicated, refined, or rejected through empirical investigation. The framework is therefore offered as a starting point for interdisciplinary dialogue rather than as a definitive account of human development.

If subsequent research supports the proposed constructs, they may contribute to a broader understanding of developmental movement before observable manifestations emerge and thereby expand opportunities for prevention, restoration, and human flourishing. If future evidence fails to support the framework, its propositions should be modified or abandoned in accordance with the principles of scientific inquiry. Such openness to empirical evaluation is essential to the continued development of the framework and to its integration within contemporary developmental science.

## 10 Conclusion

The Vilayam Framework introduces a conceptual model proposing that human development may be understood as a bidirectional process characterised by movement toward increasing integration or increasing fragmentation across the lifespan. Building upon established work in developmental psychology, positive psychology, prevention science, and contemplative research, the framework advances three interconnected theoretical propositions: that human development is fundamentally bidirectional; that progressive internal unconsciousness while biologically alive represents a proposed developmental construct centred on conscious participation; and that the Journey Before Manifestation constitutes *a proposed developmental domain* for investigating developmental processes preceding observable manifestations.

Rather than replacing existing theories, diagnostic systems, or evidence-based interventions, the framework is intended to complement contemporary approaches by extending the scope of developmental inquiry. Its principal contribution is the proposal that developmental science may benefit from investigating not only observable manifestations and recognised risk factors, but also the developmental trajectories through which manifestations may emerge.

As a conceptual framework, the Vilayam model does not claim empirical validation. Instead, it offers a set of theoretically defined propositions intended to stimulate interdisciplinary dialogue and future scientific investigation. The framework’s long-term value will depend upon whether its constructs can be operationalised, measured, compared with existing theories, and evaluated through rigorous empirical research.

If future investigations support the framework’s propositions, they may contribute to earlier recognition of developmental change, broaden opportunities for prevention and restoration, and enrich contemporary understanding of human flourishing. If empirical evidence does not support these propositions, the framework should be refined or rejected in accordance with the principles of scientific inquiry. In either case, the framework seeks to contribute to ongoing efforts to expand knowledge of human development through open, critical, and evidence-informed investigation.

## Data Availability

The original contributions presented in the study are included in the article/supplementary material, further inquiries can be directed to the corresponding author.
